# Measuring Diabetic Medication Adherence and Factors That Lead to Non-adherence Among Patients in Erbil

**DOI:** 10.7759/cureus.70397

**Published:** 2024-09-28

**Authors:** Salih Al-Chawishli, Kawa Dizaye, Suha Azeez

**Affiliations:** 1 Therapeutics, Kurdistan Higher Council of Medical Specialties, Erbil, IRQ; 2 Therapeutics and Medical Pharmacology, College of Medicine, Hawler Medical University, Erbil, IRQ; 3 Therapeutics, College of Pharmacy, Hawler Medical University, Erbil, IRQ

**Keywords:** adherence, diabetes, erbil, mmas-8, questionnaire

## Abstract

Introduction

T2D is a chronic and progressive disorder characterized by persistent hyperglycemia resulting from inadequate insulin secretion or utilization. The global prevalence of T2D is increasing rapidly, posing a significant health burden in many regions. In the Kurdistan region of Iraq, T2D presents a significant health burden, exacerbated by socioeconomic changes, dietary shifts, and rising obesity rates. Poor adherence to antidiabetic medications is a major factor contributing to poor glycemic control, accelerating disease progression, and increasing complications. This study aims to assess medication adherence rates among adult T2D patients in Erbil using the Kurdish version of the Morisky Medication Adherence Scale-8 (MMAS-8) and identify factors associated with non-adherence.

Methods

We conducted a cross-sectional study at public and private clinics in Erbil City, Kurdistan, Iraq, between May 1 and September 30, 2023. A convenience sample of 300 adult Kurdish T2D patients, aged ≥ 25 years and on antidiabetic medications for three months or more, was recruited. Data were collected using a structured questionnaire comprising sociodemographic characteristics, clinical and anthropometric measures, and medication adherence assessed by the Kurdish version of the MMAS-8. Statistical analysis included analysis of variance, Kruskal-Wallis, chi-square, and logistic regression models to identify factors associated with medication adherence.

Results

Of the 300 participants, 81 (27%) demonstrated high adherence, 98 (32.6%) moderate adherence, and 121 (40.3%) low adherence based on the MMAS-8. Low adherence was significantly associated with lower education (56/121, 46.3% vs. 13/81, 16.0%, p < 0.001), unemployment (73/121, 60.3% vs. 29/81, 35.8%, p = 0.008), rural residence (41/121, 33.9% vs. 10/81, 12.3%, p < 0.001), and lower income (62/121, 51.2% vs. 12/81, 14.8%, p < 0.001). High adherence was linked to better diabetes knowledge, home glucose monitoring, and exercise. High adherence was also associated with better glycemic control, with 76/81 (93.8%) of highly adherent patients achieving glycated hemoglobin (HbA1c) <7%, compared to 15/121 (12.4%) in the low adherence group (p < 0.001). Multivariate analysis identified HbA1c, dyslipidemia, and home blood glucose monitoring as independent factors associated with high adherence.

Conclusions

This study highlights the substantial impact of socioeconomic, behavioral, and clinical factors on medication adherence among T2D patients in Erbil. Low adherence is associated with lower education, income, and awareness of diabetes management, while high adherence is linked to improved glycemic control and reduced complications. Targeted interventions addressing these factors are essential to enhance adherence and optimize T2D management in this population.

## Introduction

Type 2 diabetes (T2D) is a chronic, progressive disorder characterized by persistent hyperglycemia resulting from inadequate insulin secretion or impaired insulin utilization [1]. The global prevalence of diabetes is rising sharply, with projections suggesting an increase from 529 million cases in 2021 to approximately 1.3 billion by 2050 [2]. In the Kurdistan region of Iraq, T2D poses a significant health burden, leading to high rates of disability and economic hardship. These challenges are driven by rapid socioeconomic changes, shifts in dietary patterns, and rising obesity rates [3]. 

T2D is associated with severe acute and chronic complications, both microvascular and macrovascular, leading to increased morbidity and premature mortality [4]. Poor adherence to antidiabetic medications is a major factor contributing to inadequate glycemic control in patients with T2D, accelerating disease progression and increasing the risk of severe complications, such as retinopathy, nephropathy, neuropathy, cardiovascular disease, and mortality [5]. Despite the wide range of available oral antidiabetic drugs, achieving optimal blood glucose control (defined as glycated hemoglobin or HbA1c below 7.0%) remains a significant challenge for patients with T2D, largely due to poor adherence [6].

Multiple factors may influence medication non-adherence among T2D patients, including patient characteristics (e.g., sex, age, socioeconomic status, education level), treatment cost and access, patient beliefs and motivations, treatment adverse effects, lack of social support, poor patient-provider communication, and forgetfulness [7-9]. Understanding these factors is essential for designing targeted interventions to improve adherence and patient outcomes. 

Accordingly, this study aims to assess medication adherence rates among adult T2D patients in Erbil using the validated Kurdish version of the Morisky Medication Adherence Scale-8 (MMAS-8) and identify potential predictors of non-adherence, including social, cultural, and economic factors, to optimize management strategies for better glycemic control.

## Materials and methods

We conducted a cross-sectional study at two centers in Erbil City, Kurdistan Region, Iraq, one public clinic (Layla Qasim) and one private clinic. Data were gathered between May 1 and September 30, 2023. We randomly selected visitors to both clinics to form a convenience sample of patients. We explained the study's goals to each patient individually. Patients were asked if they had been using any diabetes medications for longer than three months. Only those who expressed interest in participating in the study and provided verbal informed consent were enrolled. We guaranteed patients anonymity and the confidentiality of their responses to the questionnaire. The Kurdistan Higher Council of Medical Specialities Research Protocol Ethics Committee approved the study design (approval no. 976).

The questionnaire consisted of three sections, and data were collected using an iPad. Part one covered the participants' sociodemographic characteristics, including age, sex, occupation, educational status, marital status, and monthly income. Part two included anthropometric measures and clinical characteristics, such as body mass index (BMI), duration of T2D, pattern, and frequency of diabetic medications, presence of diabetic complications, other comorbidities, and laboratory findings. Part three assessed patient adherence using the MMAS-8 [3]. Each interview took approximately 20 minutes and was conducted by the investigator, a pharmacist. A validated Kurdish version of the MMAS-8 was used to measure adherence [3].

Eligible participants were adult Kurdish outpatients (aged ≥ 25 years) who had been diagnosed with and treated for T2D and had been taking their medication(s) for three months or more. Our sample included patients treated for T2D who may have presented with other chronic comorbidities. We excluded newly diagnosed T2D patients and those who refused to participate.

Based on a previous similar study [10], we estimated a required sample size of 298 patients, assuming a medication adherence rate of 74.6% among patients with T2D, a margin of error of 5%, a power of 80%, an alpha of 0.05, and an attrition rate of 2%. We calculated the sample size using the 'pwr' package of R software (version 4.3.1, R Foundation for Statistical Computing, Vienna, Austria).

Statistical analysis

We summarized participant characteristics using the mean and standard deviation for continuous variables and frequency and percentage for categorical variables. We made between-group comparisons based on medication adherence levels using analysis of variance or Kruskal-Wallis tests for continuous factors and chi-square or Fisher's exact tests for categorical factors. For continuous variables, we chose between parametric and non-parametric tests for comparison based on the results of the normality check conducted using the Shapiro-Wilk test.

We constructed univariate and multivariate logistic regression models to identify factors associated with high medication adherence. Variables with p < 0.05 in univariate testing were entered into the multivariate model. We calculated odds ratios (OR) and 95% confidence intervals (CI). We assessed the final model using the area under the receiver operating characteristic curve (AUROC). Two-tailed p-values < 0.05 were considered statistically significant. All statistical analyses were performed using R version 4.3.1 (R Foundation for Statistical Computing, Vienna, Austria).

## Results

Baseline characteristics

Table 1 summarizes the baseline characteristics of the study population stratified by medication adherence level. Of the 300 participants, 81 (27%) demonstrated high adherence, 98 (32.6%) moderate adherence, and 121 (40.3%) low adherence based on the MMAS-8. There were no significant differences in mean age (p = 0.5) or family history (p = 0.11) across adherence groups. However, a significantly higher proportion of female patients than male patients exhibited low adherence (71/121, 58.7% vs. 50/121, 41.3%, p = 0.039).

**Table 1:** Comparing demographic characteristics among the participants stratified by their adherence level (N=300) using Fisher's exact test ^a^P is significant at <0.05.

Characteristics		Overall, N = 300, n (%)	High adherence, N = 81, n (%)	Moderate adherence, N = 98, n (%)	Low adherence, N = 121, n (%)	P-valuea
Age (years)	20-30	8 (2.7%)	1 (1.2%)	5 (5.1%)	2 (1.7%)	0.5
	31-40	16 (5.3%)	3 (3.7%)	4 (4.1%)	9 (7.4%)	
	41-50	60 (20.0%)	19 (23.5%)	21 (21.4%)	20 (16.5%)	
	51-60	102 (34.0%)	31 (38.3%)	28 (28.6%)	43 (35.5%)	
	60+	114 (38.0%)	27 (33.3%)	40 (40.8%)	47 (38.8%)	
Sex	Male	151 (50.3%)	46 (56.8%)	55 (56.1%)	50 (41.3%)	0.039
	Female	149 (49.7%)	35 (43.2%)	43 (43.9%)	71 (58.7%)	
Family history of diabetes		238 (79.3%)	60 (74.1%)	75 (76.5%)	103 (85.1%)	0.11

Socioeconomic factors

Participants with low adherence had lower education levels, with 56/121 (46.3%) being illiterate compared to 13/81 (16.0%) in the high adherence group (p < 0.001). Significant differences were also observed across adherence groups in employment, residence, income, medication procurement source, and ability to afford medications (p < 0.05 for all). Compared to the high adherence group, those with low adherence had higher rates of unemployment (73/121, 60.3% vs. 29/81, 35.8%, p = 0.008), rural residence (41/121, 33.9% vs. 10/81, 12.3%, p < 0.001), and monthly income less than 500,000 Iraqi dinars (62/121, 51.2% vs. 12/81, 14.8%, p < 0.001). The high adherence group was more likely to afford monthly medications (79/81, 97.5% vs. 108/121, 89.3%, p < 0.001). Co-residence status was similar between groups, with most participants living with family rather than alone (282 participants, 94%). A summary of socioeconomic factors is shown in Table 2.

**Table 2:** Comparison of socioeconomic factors among participants stratified by adherence level (N=300) using Fisher's exact test ^a^P is significant at <0.05. Abbreviation: ID, Iraqi dinar

Characteristics		Overall, N = 300, n (%)	High adherence, N = 81, n (%)	Moderate adherence, N = 98, n (%)	Low adherence, N = 121, n (%)	P-value^a^
Education	Illiterate	99 (33.0%)	13 (16.0%)	30 (30.6%)	56 (46.3%)	<0.001
	Primary	53 (17.7%)	13 (16.0%)	18 (18.4%)	22 (18.2%)	
	Secondary	70 (23.3%)	26 (32.1%)	20 (20.4%)	24 (19.8%)	
	High School	0 (0.0%)	0 (0.0%)	0 (0.0%)	0 (0.0%)	
	University	78 (26.0%)	29 (35.8%)	30 (30.6%)	19 (15.7%)	
Employment status	Unemployed	151 (50.3%)	29 (35.8%)	49 (50.0%)	73 (60.3%)	0.008
	Employee	95 (31.7%)	36 (44.4%)	32 (32.7%)	27 (22.3%)	
	Self-employee	54 (18.0%)	16 (19.8%)	17 (17.3%)	21 (17.4%)	
Residence	Rural	68 (22.7%)	10 (12.3%)	17 (17.3%)	41 (33.9%)	<0.001
	Urban	232 (77.3%)	71 (87.7%)	81 (82.7%)	80 (66.1%)	
Monthly income	< 500,000 ID	95 (31.7%)	12 (14.8%)	21 (21.4%)	62 (51.2%)	<0.001
	500,000 - 1,000,000 ID	79 (26.3%)	31 (38.3%)	28 (28.6%)	20 (16.5%)	
	> 1,000, 000 ID	126 (42.0%)	38 (46.9%)	49 (50.0%)	39 (32.2%)	
Ability to afford medication monthly		285 (95.0%)	79 (97.5%)	98 (100.0%)	108 (89.3%)	<0.001
Source of medication procurement	Pharmacy	222 (74.0%)	65 (80.2%)	78 (79.6%)	79 (65.3%)	0.021
	Public Sector	78 (26.0%)	16 (19.8%)	20 (20.4%)	42 (34.7%)	
Co-residents	Alone	18 (6.0%)	6 (7.4%)	3 (3.1%)	9 (7.4%)	0.4
	Family	282 (94.0%)	75 (92.6%)	95 (96.9%)	112 (92.6%)	

Behavioral factors

Significant differences were observed between adherence groups for several behavioral factors related to diabetes knowledge, monitoring, and lifestyle (Table 3). A higher proportion of participants with high adherence had information about their medications (73/81, 90.1%) compared to those with low adherence (62/121, 51.2%, p < 0.001). Participants with high adherence were also more likely to obtain information from physicians (55/81, 67.9% vs. 61/121, 50.4%, p < 0.001). Awareness of diabetes complications was greater in the high adherence group (75/81, 92.6%) than in the low adherence group (57/121, 47.1%, p < 0.001). A larger percentage of highly adherent participants monitored blood sugar at home (70/81, 86.4% vs. 78/121, 64.5%, p = 0.002) and exercised more than twice weekly (27/81, 33.3% vs. 2/121, 1.7%, p < 0.001). No significant differences were found between fear of insulin injections (p = 0.82) and smoking status (p = 0.82).

**Table 3:** Comparing behavioral factors among the participants stratified by their adherence level (N=300) using Fisher's exact test ^a^P is significant at <0.05.

Characteristics		Overall, N = 300, n (%)	High adherence, N = 81, n (%)	Moderate adherence, N = 98, n (%)	Low adherence, N = 121, n (%)	P-value^a^
Having information about medications		216 (72.0%)	73 (90.1%)	81 (82.7%)	62 (51.2%)	<0.001
Source of medication information	Pharmacist	19 (6.3%)	9 (11.1%)	7 (7.1%)	3 (2.5%)	<0.001
	Doctor	188 (62.7%)	55 (67.9%)	72 (73.5%)	61 (50.4%)	
	Nurse	0 (0.0%)	0 (0.0%)	0 (0.0%)	0 (0.0%)	
	Internet	13 (4.3%)	10 (12.3%)	3 (3.1%)	0 (0.0%)	
	None	80 (26.7%)	7 (8.6%)	16 (16.3%)	57 (47.1%)	
Awareness of diabetes complications		211 (70.3%)	75 (92.6%)	79 (80.6%)	57 (47.1%)	<0.001
Fear of insulin injection		55 (18.3%)	16 (19.8%)	16 (16.3%)	23 (19.0%)	0.8
Home monitoring of blood glucose		224 (74.7%)	70 (86.4%)	76 (77.6%)	78 (64.5%)	0.002
Weekly exercise frequency	Never	199 (66.3%)	36 (44.4%)	60 (61.2%)	103 (85.1%)	<0.001
	Once weekly	36 (12.0%)	9 (11.1%)	15 (15.3%)	12 (9.9%)	
	2 times/week	28 (9.3%)	9 (11.1%)	15 (15.3%)	4 (3.3%)	
	>2 times/week	37 (12.3%)	27 (33.3%)	8 (8.2%)	2 (1.7%)	
Smoking status	Yes	39 (13.0%)	9 (11.1%)	14 (14.3%)	16 (13.2%)	0.8

Disease management

A greater proportion of participants with low adherence were treated at the Layla Qasim public clinic compared to the private clinic (74/121, 61.2% vs. 47/121, 38.8%), while those with high adherence had more balanced treatment locations (42/81, 51.9% vs. 39/81, 48.1%, p < 0.001). The mean duration of diabetes was shorter in the high adherence group (10.4 ± 8.8 years) than in the low adherence group (13.5 ± 9.0 years, p = 0.002). Participants with low adherence visited clinics more frequently (more often than every six months, 95/121, 78.5%) than those with high adherence (20/81, 24.7%, p < 0.001). The mean number of chronic medications was higher in the low adherence group (4.1 ± 2.5) than in the high adherence group (2.8 ± 2.0, p < 0.001). Medication dosing frequency did not differ significantly between groups (p = 0.62), nor did the range of the number of medications (p = 0.083). Table 4 presents the disease management characteristics across the study groups, and Table 5 presents the comparison of the duration and number of chronic drugs taken across the groups.

**Table 4:** Comparing diabetic management characteristics among the participants stratified by their adherence level (N=300) ^a^P is significant at <0.05. Abbreviations: OD, once daily; BID, twice daily; TID, three times daily.

Characteristics		Overall, N = 300, n (%)	High adherence, N = 81, n (%)	Moderate adherence, N = 98, n (%)	Low adherence, N = 121, n (%)	Statistical test	P-value^a^
Clinical setting	Layla Qasim	150 (50.0%)	42 (51.9%)	34 (34.7%)	74 (61.2%)	Kruskal-Wallis rank sum	<0.001
	Private clinic	150 (50.0%)	39 (48.1%)	64 (65.3%)	47 (38.8%)		
Duration category of Type 2 diabetes	1-5 years	69 (23.0%)	29 (35.8%)	18 (18.4%)	22 (18.2%)	Kruskal-Wallis rank sum	<0.001
	6-10 years	204 (68.0%)	52 (64.2%)	79 (80.6%)	73 (60.3%)		
	>10 years	27 (9.0%)	0 (0.0%)	1 (1.0%)	26 (21.5%)		
Frequency of medication administration	OD	51 (17.0%)	14 (17.3%)	15 (15.3%)	22 (18.2%)	Fisher’s exact test	0.6
	BID	160 (53.3%)	48 (59.3%)	52 (53.1%)	60 (49.6%)		
	TID	89 (29.7%)	19 (23.5%)	31 (31.6%)	39 (32.2%)		
Frequency of clinic visits per month	< every 6 months	131 (43.7%)	61 (75.3%)	44 (44.9%)	26 (21.5%)	Kruskal-Wallis rank sum	<0.001
	> every 6 months	169 (56.3%)	20 (24.7%)	54 (55.1%)	95 (78.5%)		
Range category of chronic drugs taken	0-4 drugs	208 (69.3%)	63 (77.8%)	72 (73.5%)	73 (60.3%)	Kruskal-Wallis rank sum	0.083
	5-8 drugs	85 (28.3%)	17 (21.0%)	24 (24.5%)	44 (36.4%)		
	> 8 drugs	7 (2.3%)	1 (1.2%)	2 (2.0%)	4 (3.3%)		

**Table 5:** Comparing duration and number of chronic drugs among the participants stratified by their adherence level (N=300) ^a^P is significant at <0.05.

Characteristics	Overall, N = 300, mean ± SD	High adherence, N = 81, mean ± SD	Moderate adherence, N = 98, mean ± SD	Low adherence, N = 121, mean ± SD	P-value^a^
Diabetes duration (years)	12.8±9.0	10.4±8.8	14.0±8.9	13.5±9.0	0.002
Number of chronic drugs taken	3.5±2.3	2.8±2.0	3.4±2.1	4.1±2.5	<0.001

Diabetes complications

The prevalence of diabetes-related complications by medication adherence level is presented in Table 6. Overall, 154/300 (51.3%) of participants had no complications, while 30/300 (10.0%) had neuropathy alone, and 23/300 (7.7%) had neuropathy plus peripheral vascular disease (PVD). The proportions with specific complications differed significantly between adherence groups (p < 0.001). Notably, 52/81 (64.2%) of highly adherent participants had no complications, compared to only 45/121 (37.2%) of those with low adherence. The low adherence group had a higher prevalence of the following conditions compared to the high adherence group: neuropathy plus PVD (10/121, 8.3% vs. 5/81, 6.2%); triple vascular complications, defined as the involvement of three or more vascular systems ("neuropathy + nephropathy + retinopathy + PVD" or "neuropathy + retinopathy + PVD"): 3/121, 2.5% and 7/121, 5.8% vs. 1/81, 1.2% and 3/81, 3.7%); and other complication combinations (30/121, 24.8% vs. 2/81, 2.5%).

**Table 6:** Comparing the incidence of diabetes complications among the participants stratified by their adherence level (N=300) using Fisher's exact test ^a^P is significant at <0.05. Abbreviations: DFI, diabetic foot infection; PVD, peripheral vascular disease; T2D, type 2 diabetes.

T2D complications	Overall, N = 300, n (%)	High adherence, N = 81, n (%)	Moderate adherence, N = 98, n (%)	Low adherence, N = 121, n (%)	P-value^a^
None	154 (51.3%)	52 (64.2%)	57 (58.2%)	45 (37.2%)	<0.001
Neuropathy only	30 (10.0%)	11 (13.6%)	5 (5.1%)	14 (11.6%)	
Nephropathy only	4 (1.3%)	2 (2.5%)	2 (2.0%)	0 (0.0%)	
Retinopathy only	6 (2.0%)	1 (1.2%)	3 (3.1%)	2 (1.7%)	
DFI only	4 (1.3%)	0 (0.0%)	1 (1.0%)	3 (2.5%)	
PVD only	6 (2.0%)	2 (2.5%)	3 (3.1%)	1 (0.8%)	
Neuropathy + Nephropathy	4 (1.3%)	1 (1.2%)	3 (3.1%)	0 (0.0%)	
Neuropathy + PVD	23 (7.7%)	5 (6.2%)	8 (8.2%)	10 (8.3%)	
Neuropathy + Nephropathy + Retinopathy + PVD	5 (1.7%)	1 (1.2%)	1 (1.0%)	3 (2.5%)	
Neuropathy + Retinopathy + PVD	12 (4.0%)	3 (3.7%)	2 (2.0%)	7 (5.8%)	
Retinopathy + DFI + PVD	10 (3.3%)	1 (1.2%)	3 (3.1%)	6 (5.0%)	
Other	42 (14.0%)	2 (2.5%)	10 (10.2%)	30 (24.8%)	

Diabetes medications

Metformin monotherapy was the most common treatment overall (72/300, 24.0%), followed by metformin plus dipeptidyl peptidase-4 inhibitors (40/300, 13.3%). Insulin alone or in combination with metformin was used by 82/300 (27.4%) of participants (Table 7). Significant differences existed between adherence groups (p = 0.008). Participants with high adherence were more likely to use metformin alone (29/81, 35.8% vs. 24/121, 19.8% in the low adherence group), while those with low adherence more frequently used insulin-containing regimens, including metformin plus insulin (18/121, 14.9% vs. 4/81, 4.9% in the high adherence group) and metformin plus sodium-glucose cotransporter-2 inhibitors (18/121, 14.9% vs. 4/81, 4.9% in the high adherence group).

**Table 7:** Comparing the incidence of diabetes medications among the participants stratified by their adherence level (N=300) using Fisher's exact test ^a^P is significant at <0.05. Abbreviations: DPP4i, dipeptidyl peptidase-4 inhibitor; SGLT2i, sodium-glucose cotransporter-2 inhibitor; SU, sulfonylurea; T2D, type 2 diabetes; TZD, thiazolidinedione.

T2D medications	Overall, N = 300, n (%)	High adherence, N = 81, n (%)	Moderate adherence, N = 98, n (%)	Low adherence, N = 121, n (%)	P-value^a^
Metformin only	72 (24.0%)	29 (35.8%)	19 (19.4%)	24 (19.8%)	0.008
SU only	7 (2.3%)	1 (1.2%)	3 (3.1%)	3 (2.5%)	
Insulin only	50 (16.7%)	6 (7.4%)	20 (20.4%)	24 (19.8%)	
Metformin+ insulin	32 (10.7%)	4 (4.9%)	10 (10.2%)	18 (14.9%)	
Metformin + SGLT2i	29 (9.7%)	4 (4.9%)	7 (7.1%)	18 (14.9%)	
Metformin + DPP4i	40 (13.3%)	13 (16.0%)	15 (15.3%)	12 (9.9%)	
Metformin + DPP4i + SU	20 (6.7%)	6 (7.4%)	8 (8.2%)	6 (5.0%)	
Metformin + DPP4i + TZD	8 (2.7%)	3 (3.7%)	4 (4.1%)	1 (0.8%)	
Metformin +SU + SGLT2i	6 (2.0%)	1 (1.2%)	1 (1.0%)	4 (3.3%)	
Metformin + TZD	6 (2.0%)	1 (1.2%)	4 (4.1%)	1 (0.8%)	
Other regimens	30 (10.0%)	13 (16.0%)	7 (7.1%)	10 (8.3%)	

Medication adverse effects

Overall, 170/300 (56.7%) participants reported no adverse effects (Table 8). The most common adverse effects were hypoglycemia plus dizziness (43/300, 14.3%) and gastrointestinal discomfort plus metallic taste (26/300, 8.7%). A higher percentage of participants with high adherence reported no adverse effects than those with low adherence (64/81, 79.0% vs. 54/121, 44.6%, p < 0.001). Conversely, those with low adherence more often reported hypoglycemia (13/121, 10.7% vs. 3/81, 3.7%) and hypoglycemia plus dizziness (27/121, 22.3% vs. 3/81, 3.7%).

**Table 8:** Comparing the incidence of medication adverse effects among the participants stratified by their adherence level (N=300) using the Fisher's exact test ^a^P is significant at <0.05. Abbreviation: GI, gastrointestinal.

Adverse effect	Overall, N = 300, n (%)	High adherence, N = 81, n (%)	Moderate adherence, N = 98, n (%)	Low adherence, N = 121, n (%)	P-value^a^
None	170 (56.7%)	64 (79.0%)	52 (53.1%)	54 (44.6%)	<0.001
Hypoglycemia	21 (7.0%)	3 (3.7%)	5 (5.1%)	13 (10.7%)	
Weight gain	5 (1.7%)	0 (0.0%)	4 (4.1%)	1 (0.8%)	
GI discomfort	0 (0.0%)	0 (0.0%)	0 (0.0%)	0 (0.0%)	
Hypoglycemia + dizziness	43 (14.3%)	3 (3.7%)	13 (13.3%)	27 (22.3%)	
GI discomfort + metallic taste	26 (8.7%)	6 (7.4%)	12 (12.2%)	8 (6.6%)	
Other	35 (11.7%)	5 (6.2%)	12 (12.2%)	18 (14.9%)	

Comorbidities

Table 9 presents the prevalence of comorbid conditions by medication adherence level. Overall, 118/300 (39.3%) of participants had no comorbidities, while hypertension (HTN) alone (27/300, 9.0%), HTN plus dyslipidemia (23/300, 7.7%), and other conditions (88/300, 29.3%) were the most common. The distribution of comorbidities did not differ significantly between adherence groups (p = 0.11). However, some trends were noted. Highly adherent participants more often had dyslipidemia alone (6/81, 7.4% vs. 1/121, 0.8% in the low adherence group), while those with low adherence more frequently had HTN plus ischemic heart disease (9/121, 7.4% vs. 1/81, 1.2%) and other comorbidity combinations (43/121, 35.5% vs. 20/81, 24.7%).

**Table 9:** Comparing the incidence of comorbid conditions among the participants stratified by their adherence level (N=300) ^a^P is significant at <0.05. Abbreviations: IHD, ischemic heart disease; HTN, hypertension.

Comorbidities	Overall, N = 300, n (%)	High adherence, N = 81, n (%)	Moderate adherence, N = 98, n (%)	Low adherence, N = 121, n (%)	P-value^a^
None	118 (39.3%)	34 (42.0%)	37 (37.8%)	47 (38.8%)	0.11
HTN	27 (9.0%)	8 (9.9%)	13 (13.3%)	6 (5.0%)	
Dyslipidemia	8 (2.7%)	6 (7.4%)	1 (1.0%)	1 (0.8%)	
HTN + hypothyroidism	4 (1.3%)	1 (1.2%)	1 (1.0%)	2 (1.7%)	
HTN + hypothyroidism + dyslipidemia	4 (1.3%)	0 (0.0%)	2 (2.0%)	2 (1.7%)	
HTN + dyslipidemia	23 (7.7%)	6 (7.4%)	9 (9.2%)	8 (6.6%)	
HTN + dyslipidemia + IHD	18 (6.0%)	5 (6.2%)	4 (4.1%)	9 (7.4%)	
HTN + IHD	4 (1.3%)	1 (1.2%)	3 (3.1%)	0 (0.0%)	
HTN + arrhythmia	6 (2.0%)	0 (0.0%)	3 (3.1%)	3 (2.5%)	
Other	88 (29.3%)	20 (24.7%)	25 (25.5%)	43 (35.5%)	

Reasons for non-adherence based on MMAS-8 items

Significant differences were found in all items of the MMAS-8 when compared across adherence levels (all p < 0.001). The low adherence group had higher rates of forgetting medications (104/121, 86.0%), stopping medications without medical consultation (70/121, 57.9%), feeling hassled by treatment (88/121, 72.7%), and difficulty remembering doses (53/121, 43.8%), compared to the high adherence group (all 0/81, 0%). The mean MMAS-8 score for all participants was 5.8 ± 2.0, with the highest score observed in the high adherence group (8.0 ± 0), followed by the moderate adherence group (6.6 ± 0.5), and the lowest score in the low adherence group (3.7 ± 1.4, p < 0.001), indicating the scale's effectiveness in distinguishing different adherence levels. In the moderate adherence group, the most common issues were forgetting doses (35/98, 35.7%) and feeling hassled by treatment (32/98, 32.7%). However, participants with moderate adherence still exhibited higher adherence than those with low adherence across all MMAS-8 items. Table 10 summarizes participant-reported reasons for suboptimal medication adherence by adherence level.

**Table 10:** Comparing the items of the MMAS-8 scale among the participants stratified by their adherence level (N=300) using Fisher's exact test ^a^P is significant at <0.05. Abbreviation: MMAS-8, Morisky Medication Adherence Scale-8.

MMAS-8 scale item	Overall, N = 300, Yes, n (%)	High adherence, N = 81, Yes, n (%)	Moderate adherence, N = 98, Yes, n (%)	Low adherence, N = 121, Yes, n (%)	P-value^a^
Frequency of forgetting to take medication	139 (46.3%)	0 (0.0%)	35 (35.7%)	104 (86.0%)	<0.001
Non-forgetting reasons for not taking medication	59 (19.7%)	0 (0.0%)	6 (6.1%)	53 (43.8%)	<0.001
Stopping medication without consulting a doctor	80 (26.7%)	0 (0.0%)	10 (10.2%)	70 (57.9%)	<0.001
Forgetting medications during travel	111 (37.0%)	0 (0.0%)	35 (35.7%)	76 (62.8%)	<0.001
Medication adherence yesterday	267 (89.0%)	81 (100.0%)	89 (90.8%)	97 (80.2%)	<0.001
Stopping medication when feeling well	70 (23.3%)	0 (0.0%)	13 (13.3%)	57 (47.1%)	<0.001
Feeling hassled by treatment plan	120 (40.0%)	0 (0.0%)	32 (32.7%)	88 (72.7%)	<0.001
Difficulty remembering to take medication	54 (18.0%)	0 (0.0%)	1 (1.0%)	53 (43.8%)	<0.001

Metabolic parameters and glycemic control

Although BMI was comparable between groups, participants with high adherence had slightly but statistically significantly higher weights (p = 0.044) and heights (p = 0.017) than those with low adherence. Significant differences were also found in HbA1c levels (p < 0.001). The mean HbA1c was lowest in the high adherence group (6.4%) and highest in the low adherence group (9.2%). Furthermore, 76/81 (93.8%) of highly adherent participants had HbA1c levels below 7% (indicating controlled diabetes), compared to only 15/121 (12.4%) of those with low adherence (p < 0.001). Among participants with low adherence, 43/121 (35.5%) had very high HbA1c levels (>10%). Key metabolic parameters and glycemic control measures stratified by medication adherence are presented in Tables 11-12.

**Table 11:** Comparing the metabolic parameters and glycemic control among the participants stratified by their adherence level (N=300) using the Kruskal-Wallis rank sum test ^a^P is significant at <0.05. Abbreviation: HbA1c, glycated hemoglobin.

Characteristics	Overall, N = 300, mean ± SD	High adherence, N = 81, mean ± SD	Moderate adherence, N = 98, mean ± SD	Low adherence, N = 121, mean ± SD	P-value^a^
Weight (kg)	80.0±15.9	80.7±17.1	82.4±14.7	77.6±15.8	0.044
Height (cm)	165.6±13.3	168.3±10.3	165.6±17.4	163.9±10.8	0.017
Body mass index (kg/m2)	38.6±167.7	28.5±5.4	59.1±293.3	28.8±4.8	0.2
HbA1c (%)	7.8±1.8	6.4±0.6	7.2±1.0	9.2±1.9	<0.001

**Table 12:** Comparing BMI and HbA1c among the participants stratified by their adherence level (N=300) using Fisher’s exact test ^a^P is significant at <0.05. Abbreviations: BMI, body mass index; HbA1c, glycated hemoglobin.

Characteristics		Overall, N = 300, n (%)	High adherence, N = 81, n (%)	Moderate adherence, N = 98, n (%)	Low adherence, N = 121, n (%)	P-value^a^
BMI classification	Overweight	125 (41.7%)	36 (44.4%)	40 (40.8%)	49 (40.5%)	0.5
	Healthy weight	51 (17.0%)	18 (22.2%)	14 (14.3%)	19 (15.7%)	
	Obese	123 (41.0%)	27 (33.3%)	44 (44.9%)	52 (43.0%)	
	Underweight	1 (0.3%)	0 (0.0%)	0 (0.0%)	1 (0.8%)	
HbA1c category	<7%	136 (45.3%)	76 (93.8%)	45 (45.9%)	15 (12.4%)	<0.001
	7-10%	119 (39.7%)	5 (6.2%)	51 (52.0%)	63 (52.1%)	
	>10%	45 (15.0%)	0 (0.0%)	2 (2.0%)	43 (35.5%)	

Regression analysis

**Figure 1:**
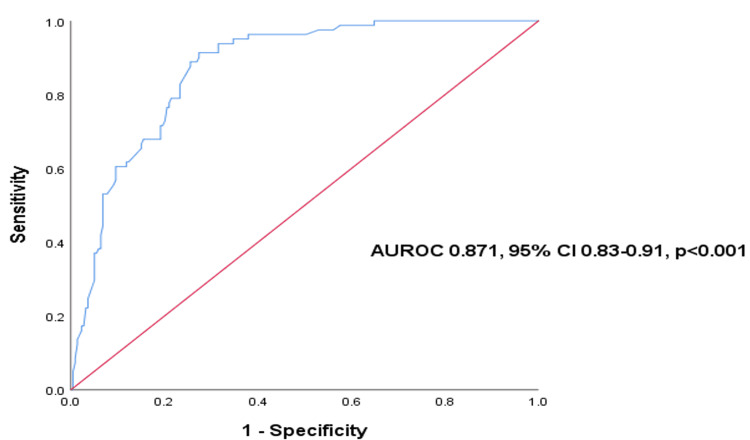
ROC Curve for the final multivariate model predicting the high adherence to antidiabetic medications (N=300). Both the area and ROC curve and the corresponding 95% confidence interval were reported in addition to the p-value. P-value is significant at <0.005. Abbreviation: AUROC, area under the receiver operating characteristic curve

In univariate analysis, high adherence was associated with several factors, including height, urban residence, higher income, employment, education level, better diabetes knowledge, self-monitoring, physical activity, fewer medications, fewer clinic visits, use of oral diabetes regimens, and presence of dyslipidemia (all p < 0.05). In contrast, longer diabetes duration, insulin therapy, and hypoglycemia were linked to lower odds of high adherence (all p < 0.05). After adjustment in multivariate modeling, HbA1c, dyslipidemia, and home blood glucose monitoring remained significantly associated with high adherence (p < 0.05). HbA1c showed the strongest association, with each 1% increase associated with a 79% decrease in the odds of high adherence (adjusted OR 0.21, 95% CI 0.10-0.39, p < 0.001). The factors associated with high medication adherence are summarized in Table 13. The AUROC curve for the final multivariate model was 0.871 (95% CI 0.83-0.97, p < 0.001), indicating strong predictive performance for predicting high adherence (Figure 1).

**Table 13:** Logistic regression analysis for the factors predicting high adherence identified using the MMAS-8 scale (N=300) ^a^P is significant at <0.05. Abbreviations: MMAS-8, Morisky Medication Adherence Scale-8; OR, odds ratio; CI, confidence interval; AOR, adjusted odds ratio; BMI, body mass index; HbA1c, glycated hemoglobin; NE, not estimated; ID, Iraqi Dinar; OD, once daily; BID, twice daily; TID, three times daily; HTN, hypertension; IHD, ischemic heart disease; T2D, type 2 diabetes; DPP4i, dipeptidyl peptidase-4 inhibitor; SGLT2i, sodium-glucose cotransporter-2 inhibitor; SU, sulfonylurea; T2D, type 2 diabetes; TZD, thiazolidinedione; GI, gastrointestinal.

Characteristics		Univariate logistic regression			Multivariate logistic regression		
		OR	95% CI	P-value^a^	AOR	95% CI	P-value^a^
Age (years)	20-30	—	—	—	—	—	—
	31-40	1.62	0.17, 36.1	0.7	—	—	—
	41-50	3.24	0.52, 62.9	0.3	—	—	—
	51-60	3.06	0.51, 58.4	0.3	—	—	—
	60+	2.17	0.36, 41.5	0.5	—	—	—
Sex	Male	—	—	—	—	—	—
	Female	0.70	0.42, 1.17	0.2	—	—	—
Family history of diabetes	Yes	—	—	—	—	—	—
	No	1.52	0.82, 2.75	0.2	—	—	—
Weight (kg)		1.00	0.99, 1.02	0.7		—	—
Height (cm)		1.03	1.01, 1.06	0.023	1.02	0.98, 1.09	0.5
Body mass index (kg/m^2^)		0.97	0.92, 1.00	0.3	—	—	—
BMI classification	Overweight	—	—	—	—	—	—
	Healthy weight	1.35	0.67, 2.68	0.4	—	—	—
	Obese	0.70	0.39, 1.23	0.2	—	—	—
	Underweight	0.00	—	>0.9	—	—	—
HbA1c (%)		0.18	0.11, 0.29	<0.001	0.21	0.10, 0.39	<0.001
Educational level	Illiterate	—	—	—	—	—	—
	Primary	2.15	0.91, 5.10	0.079	0.41	0.08, 2.03	0.3
	Secondary	3.91	1.86, 8.56	<0.001	0.68	0.16, 2.95	0.6
	University	3.92	1.90, 8.45	<0.001	0.35	0.06, 1.95	0.2
Employment status	Unemployed	—	—	—	—	—	—
	Employee	2.57	1.44, 4.61	0.001	1.42	0.40, 5.07	0.6
	Self-employee	1.77	0.86, 3.58	0.11	0.94	0.24, 3.58	>0.9
Residence	Rural	—	—	—	—	—	—
	Urban	2.56	1.28, 5.57	0.011	—	—	—
Monthly income	< 500,000 ID	—	—	—	—	—	—
	500,000 - 1,000,000 ID	4.47	2.15, 9.82	<0.001	1.40	0.37, 5.43	0.6
	> 1,000, 000 ID	2.99	1.50, 6.33	0.003	1.74	0.37, 8.76	0.5
Ability to afford medication monthly	Yes	—	—	—	—	—	—
	No	0.40	0.06, 1.50	0.2	—	—	—
Source of medication procurement	Pharmacy	—	—	—	—	—	—
	Public Sector	0.62	0.33, 1.14	0.14	—	—	—
Co-residents	Alone	—	—	—	—	—	—
	Family	0.72	0.27, 2.14	0.5	—	—	—
Having information about medications	Yes	—	—	—	—	—	—
	No	0.21	0.09, 0.43	<0.001	0.32	0.01, 10.3	0.5
Information Source	Pharmacist	—	—	—	—	—	——
	Doctor	0.46	0.18, 1.22	0.11	1.98	0.35, 11.9	0.4
	Internet	3.70	0.83, 20.7	0.10	10.6	1.04, 135	0.054
	None	0.11	0.03, 0.34	<0.001	7.95	0.13, 539	0.3
Awareness of diabetes complications	Yes	—	—	—	—	—	—
	No	0.13	0.05, 0.29	<0.001	0.36	0.08, 1.37	0.15
Fear of insulin injection	Yes	—	—	—	—	—	—
	No	0.88	0.47, 1.72	0.7	—	—	—
Home monitoring of blood glucose	Yes	—	—	—	—	—	—
	No	0.37	0.18, 0.72	0.006	0.20	0.05, 0.65	0.010
Weekly exercise frequency	Never	—	—	—	—	—	—
	Once weekly	1.51	0.62, 3.39	0.3	1.55	0.40, 5.80	0.5
	2 times/week	2.14	0.86, 5.02	0.086	0.95	0.24, 3.57	>0.9
	>2 times/week	12.2	5.60, 28.7	<0.001	3.39	0.92, 13.7	0.073
Smoking status	Yes	—	—	—	—	—	—
	No	1.27	0.60, 2.96	0.6	—	—	—
Location of the patient	Layla Qasim	—	—		—	—	—
	Private clinic	0.90	0.54, 1.50	0.7	—	—	—
Diabetes Duration (years)		0.96	0.92, 0.99	0.006	—	—	—
Duration category of Type 2 diabetes	1-5 years	—	—	—	—	—	—
	6-10 years	0.47	0.27, 0.84	0.010	0.81	0.26, 2.63	0.7
	>10 years	0.00	NE	>0.9	0.00	NE	>0.9
Frequency of medication administration	OD	—	—	—	—	—	—
	BID	1.13	0.57, 2.34	0.7	—	—	—
	TID	0.72	0.32, 1.61	0.4	—	—	—
Frequency of clinic visits per month	< every 6 months	—	—	—	—	—	—
	> every 6 months	0.15	0.08, 0.27	<0.001	0.38	0.13, 1.04	0.063
Number of chronic drugs taken		0.81	0.71, 0.92	0.001	1.19	0.85, 1.69	0.3
Range category of chronic drugs taken	0-4 drugs	—	—	—	—	—	—
	5-8 drugs	0.58	0.31, 1.04	0.075	—	—	—
	> 8 drugs	0.38	0.02, 2.31	0.4	—	—	—
Comorbidities	None	—	—	—	—	—	—
	HTN	1.04	0.40, 2.54	>0.9	2.82	0.53, 15.3	0.2
	Dyslipidemia	7.41	1.62, 52.3	0.017	70.1	3.51, 3,211	0.016
	HTN + hypothyroidism	0.82	0.04, 6.69	0.9	0.55	0.01, 30.8	0.8
	HTN + hypothyroidism + dyslipidemia	0.00	—	>0.9	0.00	—	>0.9
	HTN + hyslipidemia	0.87	0.29, 2.30	0.8	1.52	0.26, 9.13	0.6
	HTN + hyslipidemia + IHD	0.95	0.29, 2.74	>0.9	2.41	0.18, 34.5	0.5
	HTN + IHD	0.82	0.04, 6.69	0.9	3.73	0.12, 60.8	0.4
	HTN + arrythmia	0.00	—	>0.9	0.00	—	>0.9
	Other	0.73	0.38, 1.37	0.3	1.49	0.39, 5.77	0.6
T2D medications	Metformin only	—	—	—	—	—	—
	SU only	0.25	0.01, 1.55	0.2	0.12	0.00, 3.74	0.2
	Insulin only	0.20	0.07, 0.51	0.001	0.47	0.08, 2.48	0.4
	Metformin+Insulin	0.21	0.06, 0.61	0.008	0.48	0.08, 2.65	0.4
	Metformin + SGLT2i	0.24	0.06, 0.69	0.015	0.64	0.08, 4.81	0.7
	Metformin + DPP4i	0.71	0.31, 1.59	0.4	1.24	0.30, 5.05	0.8
	Metformin + DPP4i + SU	0.64	0.21, 1.78	0.4	0.72	0.11, 4.53	0.7
	Metformin + DPP4i + TZD	0.89	0.17, 3.91	0.9	1.70	0.13, 21.9	0.7
	Metformin +SU + SGLT2i	0.30	0.02, 1.96	0.3	0.35	0.01, 6.58	0.5
	Metformin + TZD	0.30	0.02, 1.96	0.3	0.11	0.00, 2.30	0.2
	Other regimens	1.13	0.47, 2.68	0.8	2.53	0.53, 13.2	0.3
Adverse effects	None	—	—	—	—	—	—
	Hypoglycemia	0.28	0.06, 0.86	0.045	1.47	0.16, 10.9	0.7
	Weight gain	0.00	—	>0.9	0.00	—	>0.9
	Hypoglycemia + dizziness	0.12	0.03, 0.36	<0.001	0.31	0.04, 1.67	0.2
	GI discomfort + metallic taste	0.50	0.17, 1.24	0.2	0.57	0.12, 2.46	0.5
	Other	0.28	0.09, 0.69	0.011	0.25	0.03, 1.37	0.13

## Discussion

In this cross-sectional study of 300 patients with T2D, we investigated medication adherence rates using the modified MMAS-8 and analyzed factors associated with adherence to antidiabetic medications. We found significant differences in medication adherence levels based on socioeconomic factors, disease management approaches, behavioral factors, and diabetes complications. Using the validated MMAS-8, 81/300 (27%) of participants exhibited high adherence, 98/300 (32.7%) moderate adherence, and 121/300 (40.3%) low adherence. Lower adherence was associated with factors such as illiteracy, low income, rural residence, unemployment, longer duration of diabetes, insulin therapy, less frequent clinic visits, a higher medication burden, and an increased prevalence of complications. In contrast, higher adherence was linked to oral regimens, home glucose monitoring, exercise, and diabetes knowledge. After adjustment, HbA1c and dyslipidemia were the strongest independent predictors of high medication adherence. Among the MMAS-8 items, frequent forgetting to take medications was the most common reason for non-adherence (104/121, 86%), while forgetting to take medication normally or during travel was the most frequent issue among moderately adherent patients (35/98, 35.7% for both).

The World Health Organization defines medication adherence as the extent to which patients take medications as prescribed, with non-adherence defined as taking less than 80% of the prescribed treatment [11]. Studies estimate that poor medication adherence accounts for 30% to 50% of T2D treatment failures, representing a major barrier to effective disease management [4,7,12]. Self-reported questionnaires, such as the MMAS-8, are commonly used to assess medication adherence due to their low cost, flexibility, and ease of administration [13,14]. The MMAS-8 allows the classification of patients into categories of low, medium, or high adherence and has demonstrated reliability and validity across diverse chronic disease populations worldwide [14,15]. In Kurdish populations, Allela et al. recently translated and validated a Kurdish version of the MMAS-8 for assessing medication adherence among T2D patients. The Kurdish MMAS-8 showed acceptable internal consistency reliability and convergent and known-groups validity, supporting its use for measuring non-adherence in this context [3]. 

The estimated adherence rate of 60% in our study, including both moderately and highly adherent patients identified on the MMAS-8 scale, aligns with the reported prevalence of medication adherence among diabetic patients, which ranges from 38.5% to 93.1% [16]. However, our estimates are higher than the reported high adherence rates in the Middle East, which range from 38% to 41% [17]. The non-adherence rates observed in our study were also higher than those found in studies using the MMAS-8 scale to assess diabetic patients. For instance, a study involving 391 diabetic adults in Ethiopia reported a non-adherence rate of 25.4% [10]. Similarly, Ibrahim et al. found a 57% adherence rate in 600 diabetic Egyptian patients assessed using a custom-designed scoring system based on glycemic control, knowledge and skills, compliance, and physician-patient relationships [18]. The discrepancies in adherence rates between our study and others could be due to differences in methodologies for measuring patient adherence, variations in patient populations (socioeconomic factors), and different cut-off points used to define good adherence [19]. 

Our findings suggest that male patients had significantly higher adherence rates than female patients. This observation is consistent with a large pharmacy database analysis of over 200,000 patients, which found that men were more likely to demonstrate good adherence to non-insulin antidiabetic medications. However, our finding contradicts several previous studies that reported no association between sex and antidiabetic medication adherence [20-22]. A recent systematic review analyzing 51 factors influencing medication adherence in diabetic populations from 26 studies reported inconsistent effects of sex due to varying definitions of adherence and different instruments used to assess adherence [19]. The review also highlighted that differences in patients' perceptions of disease susceptibility and the perceived benefits of medication may contribute to these inconsistencies. One possible explanation for the observed sex difference is that women may face greater barriers to accessing medications and health services due to sociocultural factors or family responsibilities. 

Our findings also highlighted the critical role that socioeconomic factors, such as employment, urban residence, and higher income, play in improving diabetes medication adherence. In line with our results, a recent study of 275 T2D patients assessed adherence using a structured questionnaire and found that higher education and monthly income were positively associated with increased odds of medication adherence in multivariate logistic models [22]. This has been consistently reported in similar studies evaluating factors influencing antidiabetic patient adherence [20,23-25]. Higher education is often necessary to understand the complexities of treatment and the consequences of medication non-adherence [22]. Additionally, higher monthly income is a key factor in the consistent ability to afford and procure medications, which promotes higher adherence rates. 

Among the factors associated with good adherence, glycemic control measured by HbA1c emerged as a significant predictor. Similarly, Abebe et al. found significantly higher rates of adequate glycemic control in patients with high adherence compared to those with low adherence (58.7% vs. 20.3%, p < 0.001) [10]. In a study of 565 diabetic patients assessed using the MMAS-8, Wu et al. reported that higher MMAS-8 scores were significantly associated with lower HbA1c levels (beta = -0.115, p = 0.007) [26]. Wong et al. also noted a weak but significant negative association between MMAS-8 scores and HbA1c in their assessment of antidiabetic medication adherence in T2D patients [27]. They highlighted that while MMAS-8 scores could differentiate patients based on adherence levels, they did not predict glycemic control in clinical practice [27]. Given the higher prevalence of adequate glycemic control among highly adherent patients in our cohort (93.8% vs. 12.4% in the low adherence group, p < 0.001), it is unsurprising that a greater proportion of highly adherent patients reported significantly higher rates of home glucose self-monitoring compared to the low adherence group (86.4% vs. 64.5%, p = 0.002). Interestingly, after adjustment in the final multivariate model, glucose self-monitoring remained an independent factor associated with high adherence, regardless of glycemic control. In a cross-sectional study of 130 T2D patients, Salama et al. found that regular blood glucose monitoring (76.7%) was more common among adherent patients and was an independent factor influencing adherence, as assessed by the Measure Treatment Adherence and the Adherence to Refills and Medications Scale questionnaires [28]. Consistent with our findings, a recent systematic review also concluded that blood glucose monitoring positively affects antidiabetic medication adherence [19]. 

Another factor contributing to non-adherence to treatment was the presence of comorbid chronic diseases with diabetes. Comorbid conditions are often associated with an increased medication burden and a higher incidence of adverse effects [29]. Most current evidence from similar studies supports the negative association between comorbid conditions and adherence to antidiabetic medications [19,30,31]. For example, Yosef et al. assessed factors influencing adherence in 245 T2D patients using the Medication Adherence Reporting Scale-5. They found that the absence of comorbid conditions increased the odds of good adherence (adjusted OR = 1.49, 95% CI 1.16-4.32) [30]. 

Health literacy also showed significant differences across adherence levels in our study. Knowledge about the disease, its complications, and the adverse effects of antidiabetic medications was significantly higher in patients with high adherence compared to those with lower adherence levels. This aligns with existing evidence linking increased knowledge to better adherence [29,32-34]. Improved patient awareness is associated with increased odds of compliance with physician instructions on both medications and lifestyle modifications [19]. 

Several drug-related factors significantly differed among adherence groups. Our findings indicated that highly adherent patients had lower rates of insulin use, fewer medication side effects, and a reduced average number of chronic medications. In contrast, patients with low adherence had a significantly higher average number of daily medications and a greater prevalence of adverse effects, particularly weight gain and hypoglycemia. Consistent with these findings, a systematic review of 30 studies from 10 different regions suggested that poor adherence is significantly related to a higher incidence of side effects and polypharmacy [16]. Another systematic review of 98 studies concluded that insulin therapy is a factor that can negatively influence medication adherence in the diabetic population [35]. Several studies have consistently reported adverse effects as common reasons for non-adherence to antidiabetic therapies [19,21,22,36]. 

Based on the analysis of MMAS-8 items, we found that forgetting to take medication was the most frequent reason for non-adherence in both moderate and low-adherence groups. This finding is consistent with Shaikh et al., who assessed 132 patients with T2D using a structured survey and reported that the top reason for non-adherence was forgetting to take medication (66.7%) [21]. Similarly, Alshehri et al. found that forgetting to take medication was the most reported reason for non-adherence (67.21%) in a cross-sectional study of 387 Saudi T2D patients [37]. Wabe et al. also reported medication forgetfulness as the most common barrier to adherence (50.2%) in a cross-sectional study of 384 consecutive T2D patients evaluated in outpatient settings [36]. 

Our findings have several important clinical implications. First, clinicians should be aware of the high rates of non-adherence among Kurdish T2D patients and routinely assess adherence during consultations. Brief, validated tools like the MMAS-8 can facilitate these assessments. Individualized counseling should be provided for patients showing signs of non-adherence to identify barriers and develop tailored solutions. Second, clinicians should optimize adherence among high-risk patients with longer diabetes duration, insulin use, adverse effects, lower education, and income levels. Simplifying treatment regimens, managing side effects, providing education and skills training, and assessing affordability may help overcome adherence barriers in these groups. Third, increasing patients' awareness about diabetes complications through education and motivational interviewing should be prioritized, given the strong link between knowledge and adherence. Encouraging the use of resources and technologies to support home self-monitoring can also improve adherence. Finally, providers should collaborate with local health systems and community organizations to enhance diabetes self-management through group education programs, peer support networks, and improved medication access. A multifactorial, patient-centered approach across clinical and community settings is essential for achieving optimal adherence and health outcomes in this population.

This study has several limitations. First, medication adherence was assessed through self-reported measures, which can overestimate actual adherence due to social desirability bias [38]. Second, the cross-sectional design provides only a snapshot of adherence at one point, which may fluctuate throughout the disease. Third, we were unable to account for provider-level factors. Future studies should include more objective adherence measures, such as pharmacy refill data, pill counts, or drug levels, to provide more accurate adherence estimates. Longitudinal studies assessing changes in adherence over time could further elucidate the trajectories and patterns of non-adherence. Multicenter studies with larger, more diverse samples are needed to generalize our findings.

## Conclusions

We conducted this study to assess medication adherence rates among adult T2D patients in Erbil using the validated Kurdish version of the MMAS-8 and identify predictors of non-adherence to optimize management strategies for T2D patients. Our research found that patients with higher adherence used less insulin, experienced fewer adverse drug reactions, and took fewer chronic medications on average. Patients with low adherence had an increased prevalence of adverse effects, particularly weight gain and hypoglycemia, and had considerably more daily prescriptions than those in the high adherence group. In both the moderate and low adherence groups, medication forgetfulness was the most common cause of non-adherence. These findings suggest that targeted interventions addressing forgetfulness and medication burden, promoting adherence education, and simplifying treatment regimens are essential to improve glycemic control and reduce complications in this population.
